# Carbon Nanoparticles-Decorated Carbon Nanotubes

**DOI:** 10.1038/s41598-020-61726-4

**Published:** 2020-03-17

**Authors:** Ahmed Awadallah-F, Shaheen Al-Muhtaseb

**Affiliations:** Department of Chemical Engineering, Qatar University, P.O. Box 2713 Doha, Qatar

**Keywords:** Carbon nanotubes and fullerenes, Synthesis and processing

## Abstract

Multi walled carbon nanotubes (MWCNTs) were decorated by activated carbon nanoparticles of resorcinol-formaldehyde aerogels. Carbon nanospheres and MWCNTs were mixed by equal mass ratios for different durations. The products were characterized by Raman spectroscopy, thermal gravimetric analysis, nanoscanning electron microscopy, transmission electron microscopy and x-ray diffraction. The results indicated that a significant decoration with carbon nanoparticles occurred onto the MWCNTs.

## Introduction

Carbon nanotubes (CNTs) drew a remarkable attention from their first discovery. This is attributed to their unique features, such as mechanical properties, electric properties, thermal stability, high chemical resistance and large surface areas^1^. As a result of these characteristics, they became strong candidates for numerous applications; including catalytic processes, water treatment, drug delivery, gene transfer, transparent conducting membranes and electrochemical analysis^2–7^. The idea of CNT decoration was introduced to widen their applications in different fields. It was reported in various works of literature that it was possible to decorate CNTs with either organic compounds or metallic nanoparticles^8–10^. The importance of carbon aerogels is assigned to their hierarchical porous properties, which makes them suitable for use in numerous applications^11–14^. For example, carbon aerogels are utilized in catalyst support, separation tools, eclectic supercapacitor materials and battery construction^15,16^. It is noteworthy to mention that, to the authors’ knowledge, the carbon-carbon decoration of multiwall carbon nanotubes (MWCNTs) by carbon nanoparticles (CNp) was not tackled in literature. Therefore, in this work, we disclose our method of decorating MWCNTs with resorcinol-formaldehyde activated carbon aerogel (RFA) CNp. The hybrid carbon nanoproducts will be characterized by various devices for tracking the changes of outcome carbon nanoproducts.

## Results and Discussion

**Figure 1:**
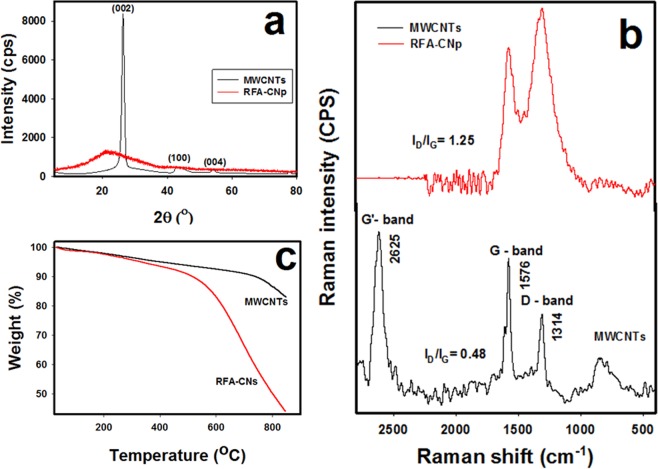
(**a**) XRD patterns, (**b**) Raman spectra and (**c**) TGA thermograms of MWCNTs (black line) RFA-CNp (red line) samples.

Figure 1(a–c) exibits the characterestics of MWCNTs and RFA-CNp via XRD, Raman spectra and TGA, respectively. Figure 1a exposes the XRD patterns of MWCNTs (black line) and RFA-CNp (red line) samples. It was observed from Fig. 1a that the characteristic bands of MWCNTs are at 2θ = 26°, 42° and 53.8°; which refer to (002), (100) and (004) reflection of the MWNTs, accdringly^17^. Moreover, RFA-CNp has no crystalline peaks as seen from Fig. 1a. The higher crystallinity in MWCNTs than RFA-CNp is due to the graphitic portion that results in the high order of MWCNTs’ structures.

Figure 1b shows the Raman spectra of MWCNTs (black line) and RFA-CNp (red line) samples. It is noteworthy to declare that the Raman spectra can be utilized to estimate the degree of defects or disorders of MWCNTs and RFA-CNp. The three fingerprint bands of pristine MWCNTs are the D-band at 1314 cm^−1^, G-band at 1576 cm^−1^ and G’-band at 2625 cm^−1^. The intensity of the D-band (I_D_) indicates to disordered or amorphous carbon portions, while that of the G band (I_G_) indicates to graphite or ordered carbon portions in the MWCNTs, and that of the G’ band indicates to a second harmonic of the D-band^18^. Moreover, the relative crystallinity can be indicated by the I_D_/I_G_ ratio. The values of I_D_/I_G_ ratio are 0.48 and 1.25 for MWCNTs and RFA-CNp, respectively. Thus, the order (crystallinity) of MWCNTs is higher than that of RFA-CNp. Figure 1c shows the TGA thermograms of MWCNTs (black line) and RFA-CNp (red line) samples. It is observed that the thermal stability of MWCNTs is higher than that of RFA-CNp. This is attributed to the presence of graphic structure that resists the thermal decomposition in MWCNTs, on contrary to the amorphous structure that has less resistance to thermal decomposition in RFA-CNp.

**Figure 2:**
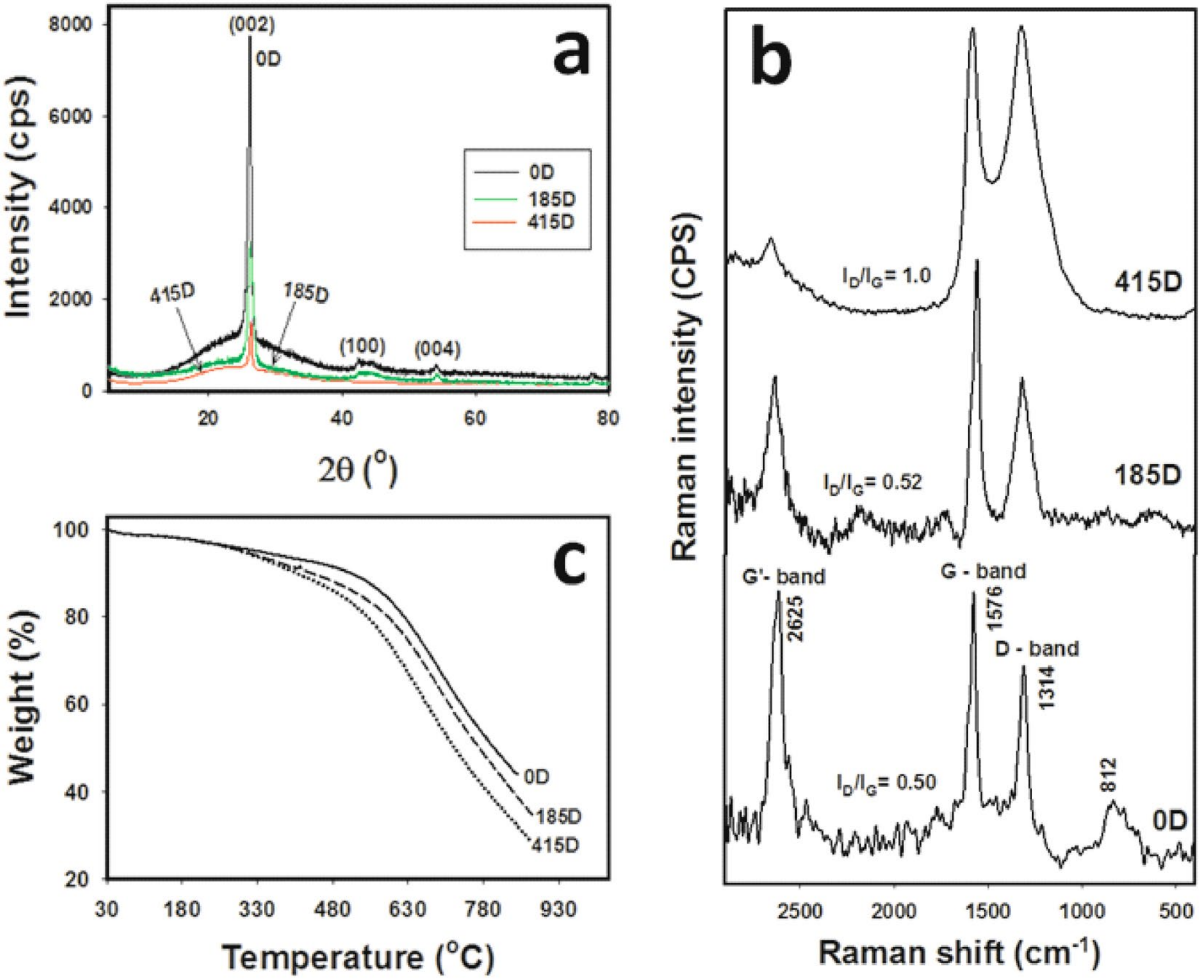
(**a**) XRD patterns, (**b**) Raman spectra and (**c**) TGA thermograms of MWCNTs/RFA-CNp hybrids with 50/50 g/g at mixing times of 0, 185 and 415 days.

Figure 2(a–c) exibits the characterestics of MWCNTs decorated with RFA-CNp via XRD, Raman spectra and TGA, accordingly; for samples decorated for various number of days as denoted by the sample codes in the form *n*D where the number *n* stands for the number of days in mixing. It is seen form the XRD patterns in Fig. 2a that the crystallinity of MWCNTs decreases by increasing the time of mixture stirring. The characteristic peaks of MWCNTs are at 2θ = 26°, 42° and 53.8°; which refer to the (002), (100) and (004) reflections of the MWCNTs, respectively^19^. The peak observed at 26° indicates to the (002) diffractions of graphite, which confirms that the hexagonal graphite structure is still in a good state in all samples^20^. The peaks at 2θ of 42.4° and 53.8° refer to the in-plane graphitic structure^21^. Furthermore, the intensities of the (002) diffraction in the samples 0D, 185D and 415D are 7774, 3229 and 1596, accordingly. Hence, the intensity of the (002) diffraction of 185D represents 41.53% of that of 0D and the intensity of 415D represents 20.52% of that of 0D. Figure 2b shows the Raman spectra of MWCNTs/RFA-CNp hybrids. Raman spectra can be utilized to assess the degree of defects or disorders of MWCNTs after decoration with RFA-CNp. Three characteristic peaks of pristine MWCNTs can be seen as the D-band at 1314 cm^−1^, G-band at 1576 cm^−1^ and G’-band at 2625 cm^−1^. The intensity of the D band (I_D_) indicates to disordered or amorphous carbon portions, while that of the G band (I_G_) refers to graphite or ordered carbon portions in the MWCNTs, and that of the G’ band indicates to a second harmonic of the D-line^22^. Moreover, the disorder/defects can be determined by the I_D_/I_G_ ratio. The values of I_D_/I_G_ ratio are 0.50, 0.52 and 1.00 for the 0D, 185D and 415D samples, accordingly. Therefore, the degree of disorder/defects of decorated CNTs decreases when increasing the mixing time. On other words, the more migration of RFA-CNp particles to the surface of MWCNTs, the disorder/defects phenomena increases.

Figure 2c exposes TGA thermograms of MWCNTs/RFA-CNp mixture. It was observed that, when increasing the mixing time, the thermal stability of MWCNTs decreases. For instance, the mass losses of the 0D, 18D5 and 415D samples at 353 °C were 5.11, 6.56 and 7.38%, respectively. Furthermore, the mass losses of 0D, 185D and 415D samples at 484 °C were 8.67, 11.93 and 13.73%, respectively. The mass losses (%) of 0, 185 and 415D at 678 °C were 29.17, 34.23 and 42.69%, respectively. Similarly, the mass losses of 0D, 185D and 415D at 842 °C were 55.52, 61.71 and 69.02%, respectively. Consequently, the presence of RFA-CNp into the matrix of MWCNTs impacts significantly the samples’ thermal stability. The migration of RFA-CNp to the surface of MWCNTs surface weakens the graphic structure of MWCNTs. XRD, Raman spectra and TGA of the utilized MWCNTs and RFA-CNp are described in the Fig. 1.

**Figure 3:**
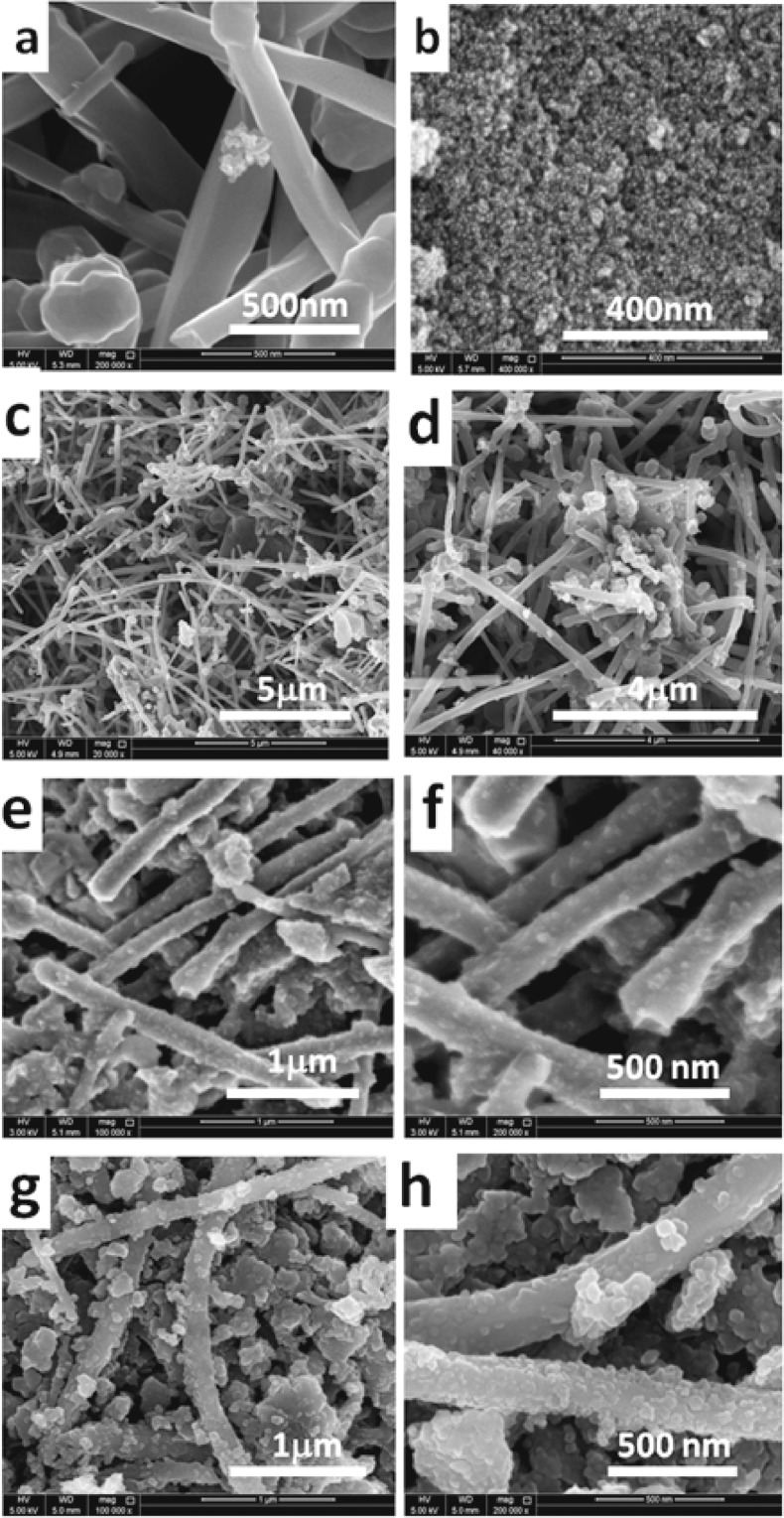
NanoSEM photomicrographs of MWCNTs/RFA-CNp with 50/50 g/g mixing ratio at different mixing times and different scales of magnifications. (**a**,**b**) refers to pristine MWCTs and CNp, respectively. Each couple of photomicrographs of (**c**,**d**) 0D, (**e**,**f**) 185D and (**g**,**h**) 415D refer to different scales of magnifications for the corresponding sample.

Figure 3(a–h) exhibits the NanoSEM photomicrographs of MWCNTs/RFA-CNp at variable times of stirring; 0D, 185D and 415D. Figure 3(a,b) exposes the NanoSEM photomicrographs of MWCNTs and RFA-CNp samples, respectively. It is seen from Fig. 3a that the morphology of MWCNTs is tubular in shape. From Fig. 3b, it is observed that the morphology of RFA-CNp seems like a spherical style. On the other hand, Fig. 3(c,d) exposes the two amplifications of MWCNTs/RFA-CNp at 0D. It can be noticed that the morphology MWCNTs are in good state and that of RFA-CNp also remains well. Figure 3(e,f) shows the two amplifications of MWCNTs/RFA-CNp of 185D at 1μm and 500 nm, accordingly. It can be observed from Fig. 3(e,f) that the change appeared clearly on the outer surface morphology of MWCNTs, which becomes decorated with RFA-CNp. Figure 3(g,h) shows the two amplifications of MWCNTs/RFA-CNp of 415D at 1μm and 500 nm, accordingly. It can be noticed that the attachment RFA-CNp onto MWCNTs grows more densely and abundantly in the case of 415D. Therefore, it can be deduced that this hybrid carbon product has new features could be used in versatile applications and need further investigations.

**Figure 4:**
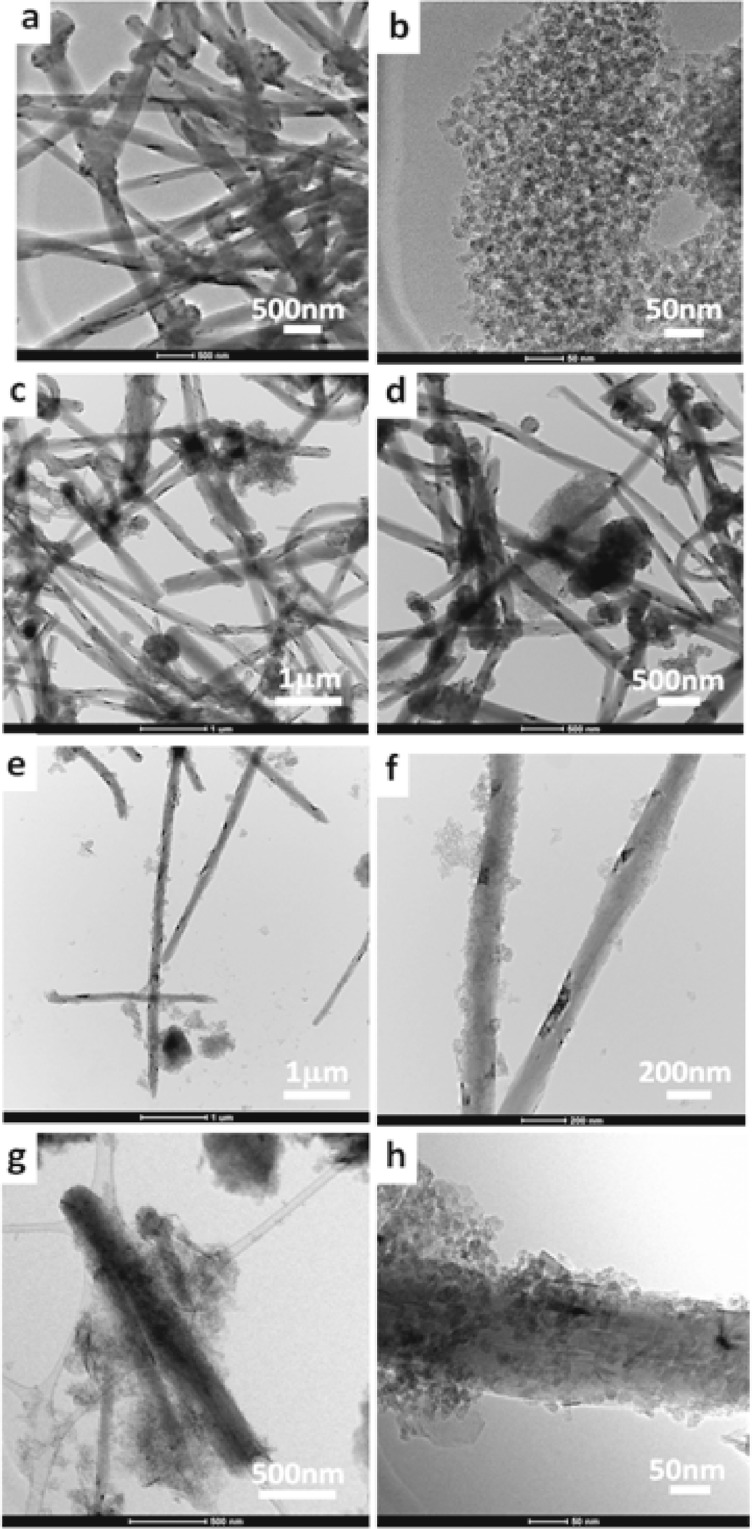
TEM photomicrographs of MWCNTs/RFA-CNp with 50/50 g/g at different aqueous mixing time of stirring and different scale of magnification. (**a**,**b**) refers to pristine MWCTs and CNp, respectively. Each couple of photomicrographs of (**c**,**d**) 0D, (**e**,**f**) 185D and (**g**,**h**) 415D refer to different scale of magnifications.

Figure 4(a–f) exposes the TEM photomicrographs of MWCNTs/RFA-CNp at variable times of stirring; 0D, 185D and 415D. Figure 4(a,b) shows the TEM photomicrographs of MWCNTs and RFA-CNs samples, accordingly. It can be confirmed again that the morphological shapes of MWCNTs and RFA-CNp are tubular and seemingly spherical style, accordingly. Figure 4(c,d) shows the MWCNTs/RFA-CNp of 0D at two magnifications; 1μm and 500 nm, respectively. It is observed from both magnifications that the morphologies of both of the MWCNTs and RFA-CNp remain well intact. Figure 4(e,f) exhibits the MWCNTs/RFA-CNs after stirring of 185D at two magnifications; 1μm and 500 nm, respectively. Through the observation it can be deduced that the MWCNTs become decorated with RFA-CNp. Figure 4(g,h) shows the MWCNTs/RFA-CNp of 415D at two magnifications; 1μm and 500 nm, respectively. It can be noticed from both photos that the MWCNTs become decorated densely and abundantly with RFA-CNp. Overall observation, the presence of RFA-CNp onto the surface of MWCNTs led to new decorated carbon-carbon products with different features than the pristine MWCNTs or RFA-CNp.

## Conclusion

The carbon-carbon decoration, specifically MWCNTs with carbon nanoparticles, is a novel trend that can lead to expanding the potential applications of such nanomaterials. This work is just a trial to confirm that it is possible to decorate MWCNTs with carbon aerogel nanoparticles (RFA-CNp) as an example. This is done by mixing in methanol for long durations at a relatively high temperature. Various characterization techniques; including XRD, Raman spectra, TGA, NanoSEM and TEM were utilized to investigate the decorated products. The results referred to significant changes in the properties of pristine MWCNTs and RFA-CNp, which confirmed the formation of hybrid carbon nanoproducts. Moreover, in comparison to the pristine MWCNTs or RFA-CNp, the produced hybrid carbon nanoproducts are featured with unique morphologies, crystallinities and extents of order/defects in their structures. Overall, it was found that mixing for 185 days resulted in decorating the MWCNTs with RFA-CNp to a noticeable extent, whereas mixing for 415 days resulted in a significant (and in an abundance of) decoration. These hybrid carbon nanoproducts could be used in numerous applications and are worthy of further investigations.

## Experimental section

### Materials

MWCNTs (purity > 90%, with a diameter of 110–170 nm and length of 5–9 μm, resorcinol (purity 99%) and formaldehyde (containing 10–15% methanol as stabilizer, 37 wt.% in H_2_O) were purchased from Sigma-Aldrich, (Germany). Sodium carbonate anhydrous (Fisher Scientific, UK). Ultra-pure water supplied from Elix^®^70 Water Purification System from Millipore Sigma. Other reagents (Acetone, methanol, acetic acid, nitric acid and ammonium hydroxide) are of analytical reagent grade. All chemicals are utilized as-received.

### Preparation of aerogels

Aerogels were synthesised from resorcinol and formaldehyde in presence of Na_2_CO_3_ as catalyst. The pH level of the starting solution is adapted at a neutral level with HNO_3_ and NH_4_OH buffers. The quantities of resorcinol (R), Na_2_CO_3_ catalyst (C), formaldehyde (F), and water (W) that were utilized in the preparation of the aerogel were 12.44 gram, 0.0240 g, 17.40 ml, and 32.60 ml, respectively. These quantities refer to molar ratios of R:F = 0.5, R:C = 500 and R:W = 0.05. The medium temperature is controlled at 70 ± 1 °C. The gels were cured and dried by supercritical CO_2_ extraction as detailed elsewhere^23,24^. Supercritical drying is conducted by utilizing a critical point dryer (E3100 Critical Point Dryer, Quorum Technologies - Preparation for Excellence, UK).

### Preparation of activated carbon aerogels

The dried resorcinol/formaldehyde aerogels are placed in a ceramic crucible into a tube furnace (Nabertherm GmbH, Germany), while flowing a stream of nitrogen gas (1.0 × 10^2^ cm^3^/min). The tube furnace is firstly kept at ambient temperature for ½ h to make sure that air is fully replaced with nitrogen. Afterwards, the temperature of the furnace is increased up to 500 °C with a rate of 10 °C/min, kept at 500 °C for 180 min, and then let to cool down spontaneously to ambient temperature while flowing nitrogen. The outcome RF carbon aerogel is later activated within the same ceramic crucible (after cleaning thoroughly from previous residues) by switching the nitrogen gas with a carbon dioxide gas stream (1.5 × 10^2^ cm^3^/min), heating-up the sample again with a ramp of 10 °C/min up to 700 °C, keeping the sample at 700 °C for 1 h, and then cooling the product spontaneously to ambient temperature while still flowing CO_2_ gas^24,25^. The outcome sample is redeemed to be an activated carbon aerogel and is called resorcinol-formaldehyde carbon aerogel nanoparticles, which are denoted as RFA-CNp.

### Synthesis of carbon nanospheres-decorated carbon nanotubes

MWCNTs and RFA-CNp were mixed in a fixed weight proportion of 1:1 (this ratio is selected as a fair example of the amount used between the two main ingredients) in reflux with methanol while stirring for 0, 185 and 415 days at 100 °C. The identity of these samples will be called hereafter; 0D, 185D and 415D, respectively. The sample 0D refers to mixing the samples manually in a dry state. All samples are then dried at 110 °C for 3 days^25^.

## Characterization

FT-Raman spectra were estimated by a Bruker FT-Raman spectrometer of type RFS 100/S. The morphological strutures of carbon materails were invetiagted via a FEI Nova™ nanoscanning electron microscope 450. Thermogravimetric analyses (TGA) are conducted (from 30 °C to 800 °C with a heating rate of 10 °C/min) utilizing a Perkin Elmer Pyris6 TGA analyzer with a flow of nitrogen. Transmission electron microscopy of (TEM) was conducted with a FEI Tecnai G2 F20 FE-TEM. X-ray diffraction (XRD) tests are carried out by Miniflex II Benchtop XRD apparatus, manufactured by Rigaku Corporation Japan.
